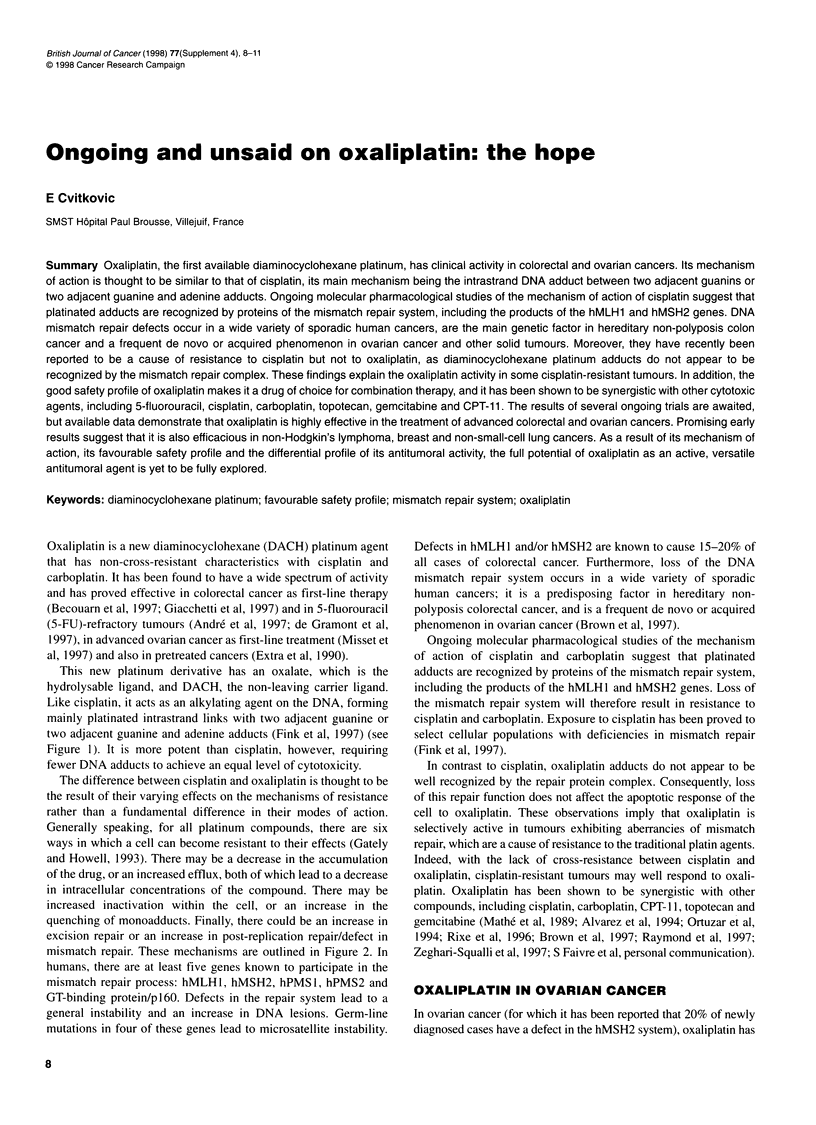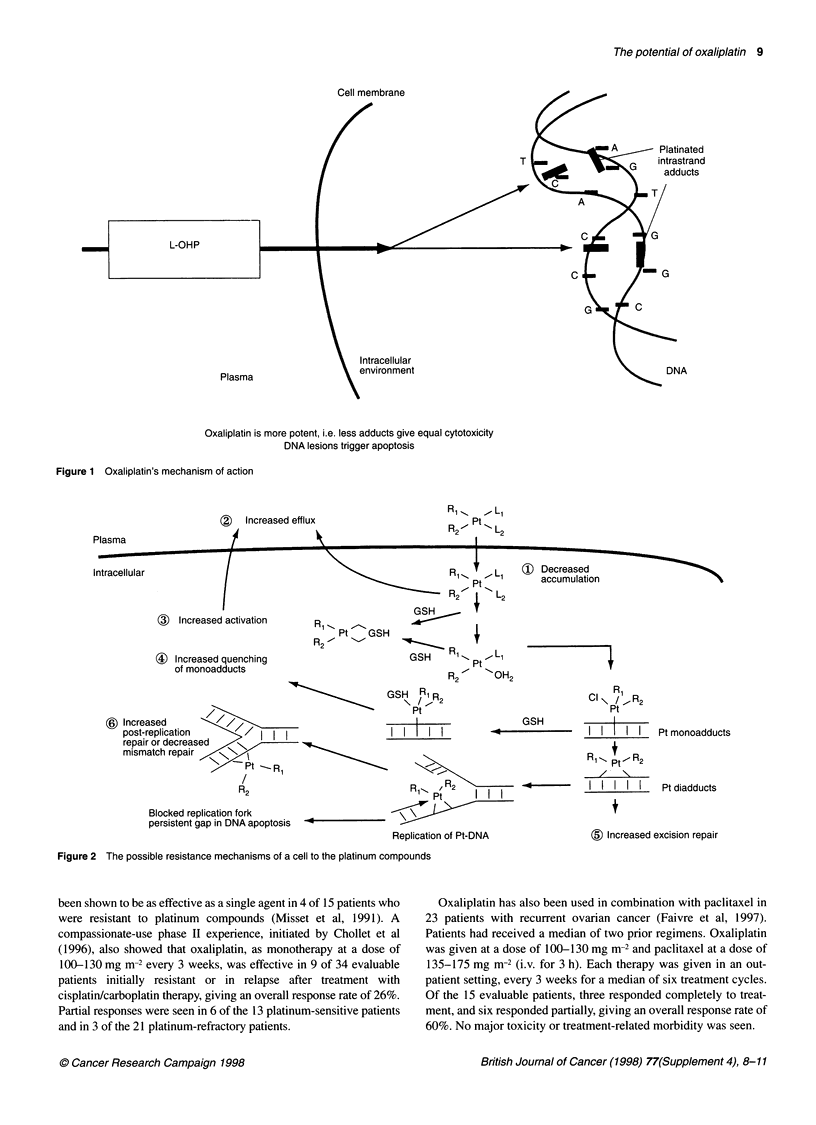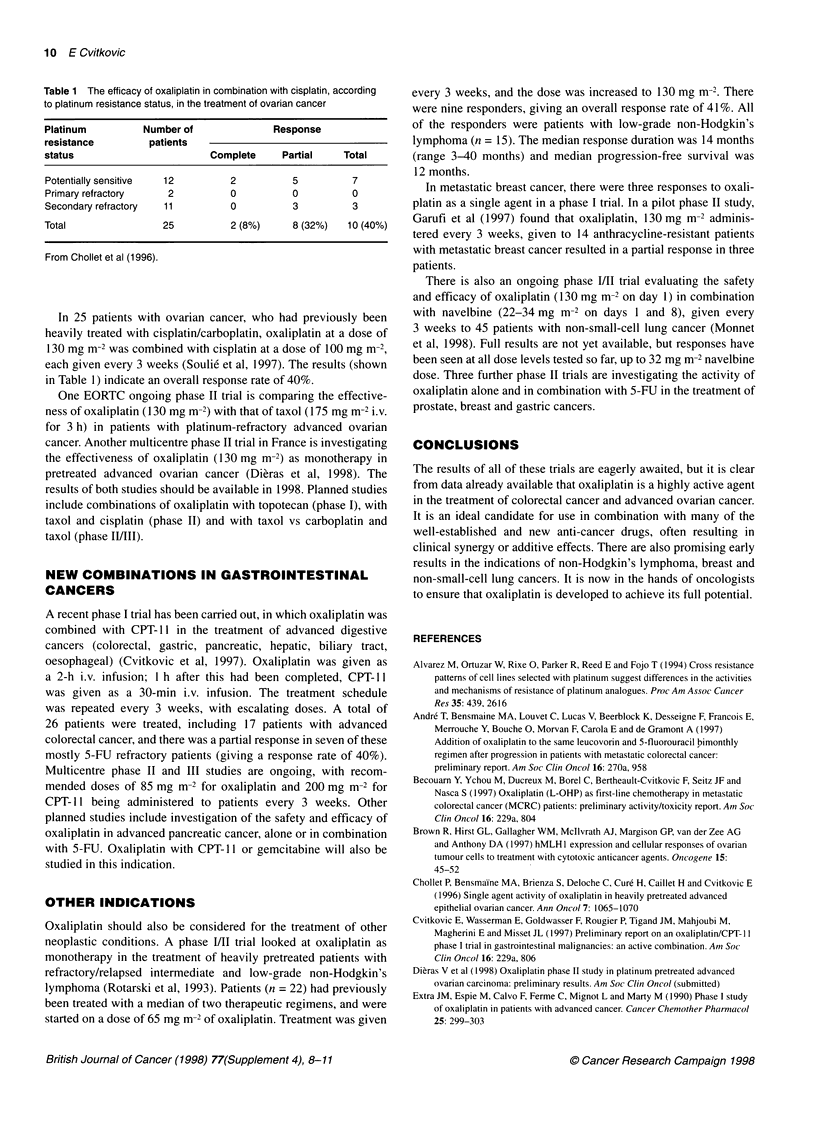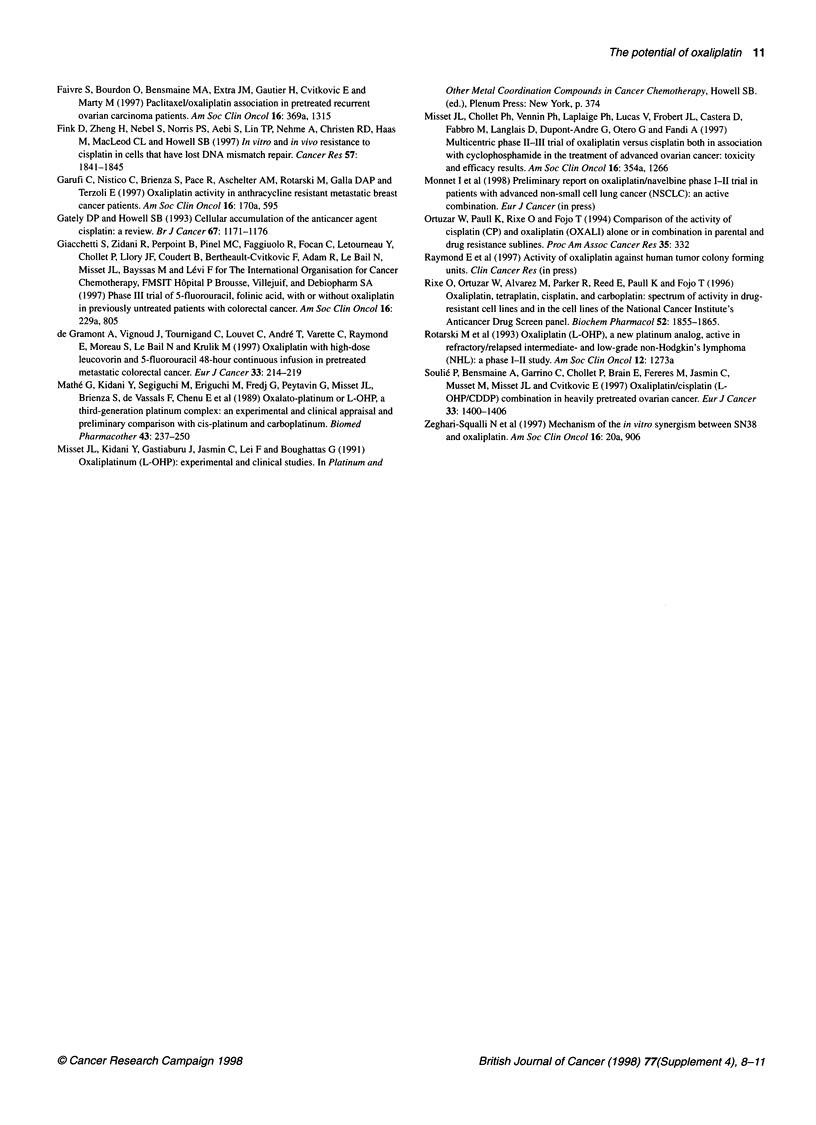# Ongoing and unsaid on oxaliplatin: the hope.

**DOI:** 10.1038/bjc.1998.429

**Published:** 1998-06

**Authors:** E. Cvitkovic

**Affiliations:** SMST HÃ´pital Paul Brousse, Villejuif, France.

## Abstract

Oxaliplatin, the first available diaminocyclohexane platinum, has clinical activity in colorectal and ovarian cancers. Its mechanism of action is thought to be similar to that of cisplatin, its main mechanism being the intrastrand DNA adduct between two adjacent guanins or two adjacent guanine and adenine adducts. Ongoing molecular pharmacological studies of the mechanism of action of cisplatin suggest that platinated adducts are recognized by proteins of the mismatch repair system, including the products of the hMLH1 and hMSH2 genes. DNA mismatch repair defects occur in a wide variety of sporadic human cancers, are the main genetic factor in hereditary non-polyposis colon cancer and a frequent de novo or acquired phenomenon in ovarian cancer and other solid tumours. Moreover, they have recently been reported to be a cause of resistance to cisplatin but not to oxaliplatin, as diaminocyclohexane platinum adducts do not appear to be recognized by the mismatch repair complex. These findings explain the oxaliplatin activity in some cisplatin-resistant tumours. In addition, the good safety profile of oxaliplatin makes it a drug of choice for combination therapy, and it has been shown to be synergistic with other cytotoxic agents, including 5-fluorouracil, cisplatin, carboplatin, topotecan, gemcitabine and CPT-11. The results of several ongoing trials are awaited, but available data demonstrate that oxaliplatin is highly effective in the treatment of advanced colorectal and ovarian cancers. Promising early results suggest that it is also efficacious in non-Hodgkin's lymphoma, breast and non-small-cell lung cancers. As a result of its mechanism of action, its favourable safety profile and the differential profile of its antitumoral activity, the full potential of oxaliplatin as an active, versatile antitumoral agent is yet to be fully explored.


*British Journal of Cancer* (1998) **77**(Supplement 4), 8–11
© 1998 Cancer Research Campaign

### Ongoing and unsaid on oxaliplatin: the hope

**E Cvitkovic**

SMST Hôpital Paul Brousse, Villejuif, France

**Summary** Oxaliplatin, the first available diaminocyclohexane platinum, has clinical activity in colorectal and ovarian cancers. Its mechanism of action is thought to be similar to that of cisplatin, its main mechanism being the intrastrand DNA adduct between two adjacent guanins or two adjacent guanine and adenine adducts. Ongoing molecular pharmacological studies of the mechanism of action of cisplatin suggest that platinated adducts are recognized by proteins of the mismatch repair system, including the products of the hMLH1 and hMSH2 genes. DNA mismatch repair defects occur in a wide variety of sporadic human cancers, are the main genetic factor in hereditary non-polyposis colon cancer and a frequent de novo or acquired phenomenon in ovarian cancer and other solid tumours. Moreover, they have recently been reported to be a cause of resistance to cisplatin but not to oxaliplatin, as diaminocyclohexane platinum adducts do not appear to be recognized by the mismatch repair complex. These findings explain the oxaliplatin activity in some cisplatin-resistant tumours. In addition, the good safety profile of oxaliplatin makes it a drug of choice for combination therapy, and it has been shown to be synergistic with other cytotoxic agents, including 5-fluorouracil, cisplatin, carboplatin, topotecan, gemcitabine and CPT-11. The results of several ongoing trials are awaited, but available data demonstrate that oxaliplatin is highly effective in the treatment of advanced colorectal and ovarian cancers. Promising early results suggest that it is also efficacious in non-Hodgkin's lymphoma, breast and non-small-cell lung cancers. As a result of its mechanism of action, its favourable safety profile and the differential profile of its antitumoral activity, the full potential of oxaliplatin as an active, versatile antitumoral agent is yet to be fully explored.

**Keywords:** diaminocyclohexane platinum; favourable safety profile; mismatch repair system; oxaliplatin

Oxaliplatin is a new diaminocyclohexane (DACH) platinum agent that has non-cross-resistant characteristics with cisplatin and carboplatin. It has been found to have a wide spectrum of activity and has proved effective in colorectal cancer as first-line therapy (Becouarn et al, 1997; Giacchetti et al, 1997) and in 5-fluorouracil (5-FU)-refractory tumours (André et al, 1997; de Gramont et al, 1997), in advanced ovarian cancer as first-line treatment (Misset et al, 1997) and also in pretreated cancers (Extra et al, 1990).

This new platinum derivative has an oxalate, which is the hydrolysable ligand, and DACH, the non-leaving carrier ligand. Like cisplatin, it acts as an alkylating agent on the DNA, forming mainly platinated intrastrand links with two adjacent guanine or two adjacent guanine and adenine adducts (Fink et al, 1997) (see Figure 1). It is more potent than cisplatin, however, requiring fewer DNA adducts to achieve an equal level of cytotoxicity.

The difference between cisplatin and oxaliplatin is thought to be the result of their varying effects on the mechanisms of resistance rather than a fundamental difference in their modes of action. Generally speaking, for all platinum compounds, there are six ways in which a cell can become resistant to their effects (Gately and Howell, 1993). There may be a decrease in the accumulation of the drug, or an increased efflux, both of which lead to a decrease in intracellular concentrations of the compound. There may be increased inactivation within the cell, or an increase in the quenching of monoadducts. Finally, there could be an increase in excision repair or an increase in post-replication repair/defect in mismatch repair. These mechanisms are outlined in Figure 2. In humans, there are at least five genes known to participate in the mismatch repair process: hMLH1, hMSH2, hPMS1, hPMS2 and GT-binding protein/p160. Defects in the repair system lead to a general instability and an increase in DNA lesions. Germ-line mutations in four of these genes lead to microsatellite instability. Defects in hMLH1 and/or hMSH2 are known to cause 15–20% of all cases of colorectal cancer. Furthermore, loss of the DNA mismatch repair system occurs in a wide variety of sporadic human cancers; it is a predisposing factor in hereditary non-polyposis colorectal cancer, and is a frequent de novo or acquired phenomenon in ovarian cancer (Brown et al, 1997).

Ongoing molecular pharmacological studies of the mechanism of action of cisplatin and carboplatin suggest that platinated adducts are recognized by proteins of the mismatch repair system, including the products of the hMLH1 and hMSH2 genes. Loss of the mismatch repair system will therefore result in resistance to cisplatin and carboplatin. Exposure to cisplatin has been proved to select cellular populations with deficiencies in mismatch repair (Fink et al, 1997).

In contrast to cisplatin, oxaliplatin adducts do not appear to be well recognized by the repair protein complex. Consequently, loss of this repair function does not affect the apoptotic response of the cell to oxaliplatin. These observations imply that oxaliplatin is selectively active in tumours exhibiting aberrancies of mismatch repair, which are a cause of resistance to the traditional platin agents. Indeed, with the lack of cross-resistance between cisplatin and oxaliplatin, cisplatin-resistant tumours may well respond to oxaliplatin. Oxaliplatin has been shown to be synergistic with other compounds, including cisplatin, carboplatin, CPT-11, topotecan and gemcitabine (Mathé et al, 1989; Alvarez et al, 1994; Ortuzar et al, 1994; Rixe et al, 1996; Brown et al, 1997; Raymond et al, 1997; Zeghari-Squalli et al, 1997; S Faivre et al, personal communication).

### OXALIPLATIN IN OVARIAN CANCER

In ovarian cancer (for which it has been reported that 20% of newly diagnosed cases have a defect in the hMSH2 system), oxaliplatin has

**Figure 1** Oxaliplatin's mechanism of action

**Figure 2** The possible resistance mechanisms of a cell to the platinum compounds been shown to be as effective as a single agent in 4 of 15 patients who were resistant to platinum compounds (Misset et al, 1991). A compassionate-use phase II experience, initiated by Chollet et al (1996), also showed that oxaliplatin, as monotherapy at a dose of 100–130 mg $m^{-2}$ every 3 weeks, was effective in 9 of 34 evaluable patients initially resistant or in relapse after treatment with cisplatin/carboplatin therapy, giving an overall response rate of 26%. Partial responses were seen in 6 of the 13 platinum-sensitive patients and in 3 of the 21 platinum-refractory patients.

Oxaliplatin has also been used in combination with paclitaxel in 23 patients with recurrent ovarian cancer (Faivre et al, 1997). Patients had received a median of two prior regimens. Oxaliplatin was given at a dose of 100–130 mg $m^{-2}$ and paclitaxel at a dose of 135–175 mg $m^{-2}$ (i.v. for 3 h). Each therapy was given in an out-patient setting, every 3 weeks for a median of six treatment cycles. Of the 15 evaluable patients, three responded completely to treatment, and six responded partially, giving an overall response rate of 60%. No major toxicity or treatment-related morbidity was seen.

© Cancer Research Campaign 1998

Table 1 The efficacy of oxaliplatin in combination with cisplatin, according to platinum resistance status, in the treatment of ovarian cancer

| Platinum resistance status | Number of patients | Response | | |
|---|---|---|---|---|
| | | Complete | Partial | Total |
| Potentially sensitive | 12 | 2 | 5 | 7 |
| Primary refractory | 2 | 0 | 0 | 0 |
| Secondary refractory | 11 | 0 | 3 | 3 |
| Total | 25 | 2 (8%) | 8 (32%) | 10 (40%) |

From Chollet et al (1996).

In 25 patients with ovarian cancer, who had previously been heavily treated with cisplatin/carboplatin, oxaliplatin at a dose of 130 mg $m^{-2}$ was combined with cisplatin at a dose of 100 mg $m^{-2}$, each given every 3 weeks (Soulié et al, 1997). The results (shown in Table 1) indicate an overall response rate of 40%.

One EORTC ongoing phase II trial is comparing the effectiveness of oxaliplatin (130 mg $m^{-2}$) with that of taxol (175 mg $m^{-2}$ i.v. for 3 h) in patients with platinum-refractory advanced ovarian cancer. Another multicentre phase II trial in France is investigating the effectiveness of oxaliplatin (130 mg $m^{-2}$) as monotherapy in pretreated advanced ovarian cancer (Dièras et al, 1998). The results of both studies should be available in 1998. Planned studies include combinations of oxaliplatin with topotecan (phase I), with taxol and cisplatin (phase II) and with taxol vs carboplatin and taxol (phase II/III).

### NEW COMBINATIONS IN GASTROINTESTINAL CANCERS

A recent phase I trial has been carried out, in which oxaliplatin was combined with CPT-11 in the treatment of advanced digestive cancers (colorectal, gastric, pancreatic, hepatic, biliary tract, oesophageal) (Cvitkovic et al, 1997). Oxaliplatin was given as a 2-h i.v. infusion; 1 h after this had been completed, CPT-11 was given as a 30-min i.v. infusion. The treatment schedule was repeated every 3 weeks, with escalating doses. A total of 26 patients were treated, including 17 patients with advanced colorectal cancer, and there was a partial response in seven of these mostly 5-FU refractory patients (giving a response rate of 40%). Multicentre phase II and III studies are ongoing, with recommended doses of 85 mg $m^{-2}$ for oxaliplatin and 200 mg $m^{-2}$ for CPT-11 being administered to patients every 3 weeks. Other planned studies include investigation of the safety and efficacy of oxaliplatin in advanced pancreatic cancer, alone or in combination with 5-FU. Oxaliplatin with CPT-11 or gemcitabine will also be studied in this indication.

### OTHER INDICATIONS

Oxaliplatin should also be considered for the treatment of other neoplastic conditions. A phase I/II trial looked at oxaliplatin as monotherapy in the treatment of heavily pretreated patients with refractory/relapsed intermediate and low-grade non-Hodgkin's lymphoma (Rotarski et al, 1993). Patients ($n$ = 22) had previously been treated with a median of two therapeutic regimens, and were started on a dose of 65 mg $m^{-2}$ of oxaliplatin. Treatment was given every 3 weeks, and the dose was increased to 130 mg $m^{-2}$. There were nine responders, giving an overall response rate of 41%. All of the responders were patients with low-grade non-Hodgkin's lymphoma ($n$ = 15). The median response duration was 14 months (range 3–40 months) and median progression-free survival was 12 months.

In metastatic breast cancer, there were three responses to oxaliplatin as a single agent in a phase I trial. In a pilot phase II study, Garufi et al (1997) found that oxaliplatin, 130 mg $m^{-2}$ administered every 3 weeks, given to 14 anthracycline-resistant patients with metastatic breast cancer resulted in a partial response in three patients.

There is also an ongoing phase I/II trial evaluating the safety and efficacy of oxaliplatin (130 mg $m^{-2}$ on day 1) in combination with navelbine (22–34 mg $m^{-2}$ on days 1 and 8), given every 3 weeks to 45 patients with non-small-cell lung cancer (Monnet et al, 1998). Full results are not yet available, but responses have been seen at all dose levels tested so far, up to 32 mg $m^{-2}$ navelbine dose. Three further phase II trials are investigating the activity of oxaliplatin alone and in combination with 5-FU in the treatment of prostate, breast and gastric cancers.

### CONCLUSIONS

The results of all of these trials are eagerly awaited, but it is clear from data already available that oxaliplatin is a highly active agent in the treatment of colorectal cancer and advanced ovarian cancer. It is an ideal candidate for use in combination with many of the well-established and new anti-cancer drugs, often resulting in clinical synergy or additive effects. There are also promising early results in the indications of non-Hodgkin's lymphoma, breast and non-small-cell lung cancers. It is now in the hands of oncologists to ensure that oxaliplatin is developed to achieve its full potential.

### REFERENCES

Alvarez M, Ortuzar W, Rixe O, Parker R, Reed E and Fojo T (1994) Cross resistance patterns of cell lines selected with platinum suggest differences in the activities and mechanisms of resistance of platinum analogues. *Proc Am Assoc Cancer Res* **35**: 439, 2616

André T, Bensmaine MA, Louvet C, Lucas V, Beerblock K, Desseigne F, Francois E, Merrouche Y, Bouche O, Morvan F, Carola E and de Gramont A (1997) Addition of oxaliplatin to the same leucovorin and 5-fluorouracil bimonthly regimen after progression in patients with metastatic colorectal cancer: preliminary report. *Am Soc Clin Oncol* **16**: 270a, 958

Becouarn Y, Ychou M, Ducreux M, Borel C, Bertheault-Cvitkovic F, Seitz JF and Nasca S (1997) Oxaliplatin (L-OHP) as first-line chemotherapy in metastatic colorectal cancer (MCRC) patients: preliminary activity/toxicity report. *Am Soc Clin Oncol* **16**: 229a, 804

Brown R, Hirst GL, Gallagher WM, McIlvrath AJ, Margison GP, van der Zee AG and Anthony DA (1997) hMLH1 expression and cellular responses of ovarian tumour cells to treatment with cytotoxic anticancer agents. *Oncogene* **15**: 45–52

Chollet P, Bensmaïne MA, Brienza S, Deloche C, Curé H, Caillet H and Cvitkovic E (1996) Single agent activity of oxaliplatin in heavily pretreated advanced epithelial ovarian cancer. *Ann Oncol* **7**: 1065–1070

Cvitkovic E, Wasserman E, Goldwasser F, Rougier P, Tigand JM, Mahjoubi M, Magherini E and Misset JL (1997) Preliminary report on an oxaliplatin/CPT-11 phase I trial in gastrointestinal malignancies: an active combination. *Am Soc Clin Oncol* **16**: 229a, 806

Dièras V et al (1998) Oxaliplatin phase II study in platinum pretreated advanced ovarian carcinoma: preliminary results. *Am Soc Clin Oncol* (submitted)

Extra JM, Espie M, Calvo F, Ferme C, Mignot L and Marty M (1990) Phase I study of oxaliplatin in patients with advanced cancer. *Cancer Chemother Pharmacol* **25**: 299–303

© Cancer Research Campaign 1998

Faivre S, Bourdon O, Bensmaine MA, Extra JM, Gautier H, Cvitkovic E and Marty M (1997) Paclitaxel/oxaliplatin association in pretreated recurrent ovarian carcinoma patients. *Am Soc Clin Oncol* **16**: 369a, 1315

Fink D, Zheng H, Nebel S, Norris PS, Aebi S, Lin TP, Nehme A, Christen RD, Haas M, MacLeod CL and Howell SB (1997) *In vitro* and *in vivo* resistance to cisplatin in cells that have lost DNA mismatch repair. *Cancer Res* **57**: 1841–1845

Garufi C, Nistico C, Brienza S, Pace R, Aschelter AM, Rotarski M, Galla DAP and Terzoli E (1997) Oxaliplatin activity in anthracycline resistant metastatic breast cancer patients. *Am Soc Clin Oncol* **16**: 170a, 595

Gately DP and Howell SB (1993) Cellular accumulation of the anticancer agent cisplatin: a review. *Br J Cancer* **67**: 1171–1176

Giacchetti S, Zidani R, Perpoint B, Pinel MC, Faggiuolo R, Focan C, Letourneau Y, Chollet P, Llory JF, Coudert B, Bertheault-Cvitkovic F, Adam R, Le Bail N, Misset JL, Bayssas M and Lévi F for The International Organisation for Cancer Chemotherapy, FMSIT Hôpital P Brousse, Villejuif, and Debiopharm SA (1997) Phase III trial of 5-fluorouracil, folinic acid, with or without oxaliplatin in previously untreated patients with colorectal cancer. *Am Soc Clin Oncol* **16**: 229a, 805

de Gramont A, Vignoud J, Tournigand C, Louvet C, André T, Varette C, Raymond E, Moreau S, Le Bail N and Krulik M (1997) Oxaliplatin with high-dose leucovorin and 5-fluorouracil 48-hour continuous infusion in pretreated metastatic colorectal cancer. *Eur J Cancer* **33**: 214–219

Mathé G, Kidani Y, Segiguchi M, Eriguchi M, Fredj G, Peytavin G, Misset JL, Brienza S, de Vassals F, Chenu E et al (1989) Oxalato-platinum or L-OHP, a third-generation platinum complex: an experimental and clinical appraisal and preliminary comparison with cis-platinum and carboplatinum. *Biomed Pharmacother* **43**: 237–250

Misset JL, Kidani Y, Gastiaburu J, Jasmin C, Lei F and Boughattas G (1991) Oxaliplatinum (L-OHP): experimental and clinical studies. In *Platinum and Other Metal Coordination Compounds in Cancer Chemotherapy*, Howell SB. (ed.), Plenum Press: New York, p. 374

Misset JL, Chollet Ph, Vennin Ph, Laplaige Ph, Lucas V, Frobert JL, Castera D, Fabbro M, Langlais D, Dupont-Andre G, Otero G and Fandi A (1997) Multicentric phase II–III trial of oxaliplatin versus cisplatin both in association with cyclophosphamide in the treatment of advanced ovarian cancer: toxicity and efficacy results. *Am Soc Clin Oncol* **16**: 354a, 1266

Monnet I et al (1998) Preliminary report on oxaliplatin/navelbine phase I–II trial in patients with advanced non-small cell lung cancer (NSCLC): an active combination. *Eur J Cancer* (in press)

Ortuzar W, Paull K, Rixe O and Fojo T (1994) Comparison of the activity of cisplatin (CP) and oxaliplatin (OXALI) alone or in combination in parental and drug resistance sublines. *Proc Am Assoc Cancer Res* **35**: 332

Raymond E et al (1997) Activity of oxaliplatin against human tumor colony forming units. *Clin Cancer Res* (in press)

Rixe O, Ortuzar W, Alvarez M, Parker R, Reed E, Paull K and Fojo T (1996) Oxaliplatin, tetraplatin, cisplatin, and carboplatin: spectrum of activity in drug-resistant cell lines and in the cell lines of the National Cancer Institute's Anticancer Drug Screen panel. *Biochem Pharmacol* **52**: 1855–1865.

Rotarski M et al (1993) Oxaliplatin (L-OHP), a new platinum analog, active in refractory/relapsed intermediate- and low-grade non-Hodgkin's lymphoma (NHL): a phase I–II study. *Am Soc Clin Oncol* **12**: 1273a

Soulié P, Bensmaine A, Garrino C, Chollet P, Brain E, Fereres M, Jasmin C, Musset M, Misset JL and Cvitkovic E (1997) Oxaliplatin/cisplatin (L-OHP/CDDP) combination in heavily pretreated ovarian cancer. *Eur J Cancer* **33**: 1400–1406

Zeghari-Squalli N et al (1997) Mechanism of the *in vitro* synergism between SN38 and oxaliplatin. *Am Soc Clin Oncol* **16**: 20a, 906

© Cancer Research Campaign 1998